# Supplementary material for: Testicular hormones mediate robust sex differences in impulsive choice in rats
Source: eLife. 2020 Sep 28;9:e58604. doi: 10.7554/eLife.58604 (PMC7521924; doi:10.7554/eLife.58604)
Supplement: Transparent reporting form [file elife-58604-transrepform.docx]

***eLife’s* transparent reporting form**

We encourage authors to provide detailed information *within their submission* to facilitate the interpretation and replication of experiments. Authors can upload supporting documentation to indicate the use of appropriate reporting guidelines for health-related research (see [EQUATOR Network](http://www.equator-network.org/%20)), life science research (see the [BioSharing Information Resource](https://biosharing.org/)), or the [ARRIVE guidelines](http://www.plosbiology.org/article/info:doi/10.1371/journal.pbio.1000412) for reporting work involving animal research. Where applicable, authors should refer to any relevant reporting standards documents in this form.

If you have any questions, please consult our Journal Policies and/or contact us: [editorial@elifesciences.org](mailto:editorial@elifesciences.org).

**Sample-size estimation**

- You should state whether an appropriate sample size was computed when the study was being designed
- You should state the statistical method of sample size computation and any required assumptions
- If no explicit power analysis was used, you should describe how you decided what sample (replicate) size (number) to use

Please outline where this information can be found within the submission (e.g., sections or figure legends), or explain why this information doesn’t apply to your submission:

Group sizes were based on previous work from our lab assessing sex differences in risky decision making and executive functions (see Orsini et al., 2016 and Blaes et al., 2019 cited in text and found in the references section). A more detailed explanation can be found in the Materials and Methods section under Subjects.

**Replicates**

- You should report how often each experiment was performed
- You should include a definition of biological versus technical replication
- The data obtained should be provided and sufficient information should be provided to indicate the number of independent biological and/or technical replicates
- If you encountered any outliers, you should describe how these were handled
- Criteria for exclusion/inclusion of data should be clearly stated
- High-throughput sequence data should be uploaded before submission, with a private link for reviewers provided (these are available from both GEO and ArrayExpress)

Please outline where this information can be found within the submission (e.g., sections or figure legends), or explain why this information doesn’t apply to your submission:

The numbers of rats that were tested under each condition are provided in the Results section. There were no outliers in the datasets, and hence no data were excluded from the analyses other than for reasons due to omissions of all trials during the pre-feeding experiments or premature death due to post-surgical complications.

**Statistical reporting**

- Statistical analysis methods should be described and justified
- Raw data should be presented in figures whenever informative to do so (typically when N per group is less than 10)
- For each experiment, you should identify the statistical tests used, exact values of N, definitions of center, methods of multiple test correction, and dispersion and precision measures (e.g., mean, median, SD, SEM, confidence intervals; and, for the major substantive results, a measure of effect size (e.g., Pearson's r, Cohen's d)
- Report exact p-values wherever possible alongside the summary statistics and 95% confidence intervals. These should be reported for all key questions and not only when the p-value is less than 0.05.

Please outline where this information can be found within the submission (e.g., sections or figure legends), or explain why this information doesn’t apply to your submission:

The statistical analyses, including the specific tests used and exact group sizes, are described in Methods, Experimental Design and Statistical Analysis (as well as in the Results). Exact p values are reported in the Results. Means and SEMs for all data are reported in the Figures and Tables. We did not present raw data in most of the figures, as we believe that the line graph format of much of the primary data would require a distracting number of lines to depict each subject. We do, however, provide all of the raw data that go into these graphs in table format in the “raw data” files.

(For large datasets, or papers with a very large number of statistical tests, you may upload a single table file with tests, Ns, etc., with reference to sections in the manuscript.)

**Group allocation**

- Indicate how samples were allocated into experimental groups (in the case of clinical studies, please specify allocation to treatment method); if randomization was used, please also state if restricted randomization was applied
- Indicate if masking was used during group allocation, data collection and/or data analysis

Please outline where this information can be found within the submission (e.g., sections or figure legends), or explain why this information doesn’t apply to your submission:

Rats were received from the vendor as either male or female, and hence this was not an assignable condition. After baseline experiments, males and females were assigned to receive either sham or gonadectomy surgeries. Assignments were based on baseline matching such that the mean and variance of their choice performance was approximately equal between surgery groups within sex prior to surgery. All other experiments employed within-subjects designs. The obvious differences in appearance between male and female rats made it impossible to mask these conditions during data collection; however, the surgical conditions within each sex were not known to the experimenters.

**Additional data files (“source data”)**

- We encourage you to upload relevant additional data files, such as numerical data that are represented as a graph in a figure, or as a summary table
- Where provided, these should be in the most useful format, and they can be uploaded as “Source data” files linked to a main figure or table
- Include model definition files including the full list of parameters used
- Include code used for data analysis (e.g., R, MatLab)
- Avoid stating that data files are “available upon request”

Please indicate the figures or tables for which source data files have been provided:

The raw data that are depicted in summary form in Figures 2-7 and that were used for statistical analyses are provided in source data files corresponding to each Figure.
